# Classification of Browning on Intact Table Grape Bunches Using Near-Infrared Spectroscopy Coupled With Partial Least Squares-Discriminant Analysis and Artificial Neural Networks

**DOI:** 10.3389/fpls.2021.768046

**Published:** 2021-10-29

**Authors:** Andries J. Daniels, Carlos Poblete-Echeverría, Hélène H. Nieuwoudt, Nicolene Botha, Umezuruike Linus Opara

**Affiliations:** ^1^Department of Viticulture and Oenology, Faculty of AgriSciences, Stellenbosch University, Stellenbosch, South Africa; ^2^ARC Infruitec-Nietvoorbij, Stellenbosch, South Africa; ^3^South African Grape and Wine Research Institute, University of Stellenbosch, Stellenbosch, South Africa; ^4^Council for Scientific and Industrial Research, Modelling and Digital Science, Stellenbosch, South Africa; ^5^SARChI Postharvest Technology Research Laboratory, Africa Institute for Postharvest Technology, Faculty of AgriSciences, Stellenbosch University, Stellenbosch, South Africa; ^6^UNESCO International Centre for Biotechnology, Nsukka, Nigeria

**Keywords:** browning, intact table grape bunches, contactless scanning, near-infrared spectroscopy, partial least squares discriminant analysis, artificial neural networks

## Abstract

Table grape browning is a complex physiological disorder that occurs during cold storage. There is a need to investigate novel and innovative ways to manage the problem that hampers the progressive and sustainable growth of table grape industries. Given the complex nature of the browning phenomenon, techniques such as near-infrared (NIR) spectroscopy can be utilized for the non-destructive classification of different browning phenotypes. In this study, NIR coupled with partial least squares discriminant analysis (PLS-DA) and artificial neural networks (ANN) were used to classify bunches as either clear or as having chocolate browning and friction browning based on the spectra obtained from intact ‘Regal Seedless’ table grape bunches that were cold-stored over different periods. Friction browning appears as circular spots close to the pedicel area that are formed when table grape berries move against each other, and chocolate browning appears as discoloration, which originates mostly from the stylar-end of the berry, although the whole berry may appear brown in severe instances. The evaluation of the models constructed using PLS-DA was done using the classification error rate (CER), specificity, and sensitivity and for the models constructed using ANN, the kappa score was used. The CER for chocolate browning (25%) was better than that of friction browning (46%) for weeks 3 and 4 for both class 0 (absence of browning) and class 1 (presence of browning). Both the specificity and sensitivity of class 0 and class 1 for friction browning were not as good as that of chocolate browning. With ANN, the kappa score was tested to classify table grape bunches as clear or having chocolate browning or friction browning and showed that chocolate browning could be classified with a strong agreement during weeks 3 and 4 and weeks 5 and 6 and that friction browning could be classified with a moderate agreement during weeks 3 and 4. These results open up new possibilities for the development of quality checks of packed table grape bunches before export. This has a significant impact on the table grape industry for it will now be possible to evaluate bunches non-destructively during packaging to determine the possibility of these browning types being present when reaching the export market.

## Introduction

Exported grapes should remain intact and free of damage or defect when they reach the consumer market. Table grape browning appears mainly as a discoloration of the pulp (flesh or internal browning) and berry skin (skin or external browning) (Vial et al., 2005). This is due to a dysfunction or disruption of the cellular membranes, which allows the mixing of the enzyme polyphenol oxidase (PPO) with phenolic substrates or compounds occurring naturally in the fruit (Ferreira, 1997; Golding et al., 1998; Kruger et al., 1999). Several different phenotypes of browning, such as external, internal, low temperature, chemical, physical, and pathogenic browning have been identified by Fourie (2009). External browning is subdivided into net-like, mottled, friction, and contact browning types. Internal browning is expressed as chocolate-, water-, and glassy berry. The post-harvest treatment of grapes with methyl bromide and carbon dioxide (CO_2_) causes damage that is known as chemical browning, while abrasions and bruises are known as physical browning, and fungal infection as pathogenic browning (Fourie, 2009).

Ever since the browning phenomenon was first reported in 1989 (Wolf, 1996), it has only become more severe. Numerous studies have been conducted to try and find out what exactly is the cause of it on table grapes, but to date, nothing has shown that there is a single dominant factor that can be repeatedly linked to either internal or external browning development (Moelich, 2010). The cultivar, seasonal variations and relative amounts of individual phenolic compounds in grapes, and the phenolic distribution in the flesh and skin (Lee and Jaworski, 1989) are just some of the factors that may influence browning while the grapes are still hanging in the vineyard. Zapata et al. (1995) also could not find a correlation between real browning in red and white grapes based on the level of peroxidase activity in the grapes.

Specific macro and/or micro-nutrients and post-harvest factors, like moisture-modifying packaging material, sulfur dioxide (SO_2_), and modified atmosphere packaging (MAP) possibly influenced the occurrence of external and/or internal browning, but Burger et al. (2005) could not discover whether a possible relationship existed between them or not. A correlation between internal browning and post-harvest treatments like methyl bromide fumigation, a toxic odorless gas used to control pests of quarantine significance in both grapes and apples, was found but the clear role that glutathione, an antioxidant preventing damage to important cellular components, played in it could not be found (Liyanage et al., 1993). Even González-Barrio et al. (2005), after thoroughly explaining the chemical process of the occurrence of browning, could not show that the post-harvest treatment with UV-C light was the single cause for the browning observed in ‘Superior Seedless’ table grape.

On white seedless table grapes, the two most common kinds of external and internal browning occur in different forms- and manifest with varying profiles of development. Friction browning, for example, is a form of external browning that occurs when circular spots close to the pedicel area of table grape berries develop as a result of rolling against each other. Chocolate browning is a form of internal browning and entails a discoloration, which originates mostly from the stylar-end of the berry, although the whole berry may appear brown in severe instances (Fourie, 2009). These variations in the phenotypic manifestation of grape berry browning pose challenges for the implementation of automated and non-destructive methods for its detection and management because currently, they can only be visually detected through vigorous inspection of the bunches.

Several reports have shown that near-infrared (NIR) spectroscopy coupled with chemometric techniques such as partial least squares (PLS) and partial least squares-discriminant analysis (PLS-DA) demonstrated to be valuable and versatile tools for the simultaneous determination of an array of quantitative and qualitative parameters on the same sample (Pérez-Enciso and Tenenhaus, 2003). This includes analyzing and classifying a variety of fruit defects and diseases. Kavdir et al. (2007) used NIR spectroscopy to assess the firmness, skin, and flesh color, as well as the dry matter content of pickling cucumbers. Fu et al. (2007) demonstrated the utility of visible (VIS)-NIR spectroscopy for discriminating between pear fruits with internal brown heart defects and clear ones. Near-infrared spectroscopy was also utilized successfully to measure the microstructure-related changes that occurred because of the internal damage in apples (Clark et al., 2003). Ozanich (2001) detected moderate to severe internal disorders in apples such as water-core, internal browning, and rot while also using NIR spectroscopy. Stemming from these successful applications of NIR spectroscopy, the next logical step was to explore this technology on table grapes. This would also lay the groundwork to pursue vision-based systems or techniques in the vineyard to evaluate the quality of grapes similar to what Pothen and Nuske (2016) have already done by evaluating the color development and harvest-readiness of intact table grape bunches in the vineyard.

Partial least squares discriminant analysis is a derivative of the standard PLS regression algorithm that uses class variables instead of numeric variables (Barker and Rayens, 2003). The use of PLS-DA in previous studies has been to assess which genes are useful in discriminating between different statuses of cancer (Pérez-Enciso and Tenenhaus, 2003). Folch-Fortuny et al. (2016) used it to successfully make a distinction between healthy and infected citrus fruits. Artificial neural networks (ANN) are a machine-learning framework that attempts to mimic the learning pattern of natural biological neural networks based on their ability to “learn” throughout a training procedure exactly where inputs and a set of anticipated results are given. Artificial neural networks are typically organized in layers, and these layers are composed of interconnected nodes that contain activation functions (Ramadan et al., 2005). It is a well-established analytical tool and has been used successfully in combination with other techniques such as principal component analyses (PCA) and PLS. Baldwin et al. (2011) used it to help analyze the data obtained by electronic noses and electronic tongues for various parameters from different products. Rodríguez et al. (2010) again determined the quality control of Colombian coffee qualities and Cajka et al. (2009) confirmed the origin of honey, while Pérez-Magariño et al. (2004) classified Spanish denomination of origin rosé wines.

The aim of this study was, therefore, to scan table grape bunches of the cultivar ‘Regal Seedless’ non-destructively before and after cold storage from 0 to 6 weeks at 0°C. ‘Regal Seedless’ is one of the white seedless cultivars on which the expression of berry browning symptoms frequently occurs (Vial et al., 2005). Moelich (2010) studied the effect of delivery air temperature (DAT), as well as the duration of forced-air cooling (FAC) on the external and internal browning of cold-stored ‘Regal Seedless’ and ‘Thompson Seedless’. He found that the berry browning index of ‘Regal Seedless’ was much higher than that of ‘Thompson Seedless’ in the different populations of the two cultivars. An investigation by Avenant (2017) on the use of gibberellic acid (GA3) and N-(2-Chloro-4-pyridyl)-N-phenylurea (CPPU) treatments to reduce or eliminate browning on ‘Regal Seedless’ found that the application of CPPU (alone or in combination with GA3) to decrease the internal browning of ‘Regal Seedless’ could not be justified. Thus, given this high susceptibility of ‘Regal Seedless’ to browning, it was a model cultivar to use in this study. Therefore, the visual assessment of the ‘Regal Seedless’ bunches occurred after a contactless scanning with the MATRIX-F instrument (Bruker Optics, Ettlingen, Germany) for various defects and browning phenotypes including chocolate and friction browning. Since individual berries behave like individual experimental units although they are part of a bunch, it was an important objective that the whole bunches must be investigated (table grapes are exported as whole bunches and not individual berries). After recording the presence (1) or absence (0) of the browning phenotypes, the spectra of the grapes, as well as the column that indicated if there was a defect or not, were combined into one large dataset. The data was then analyzed using PLS-DA and ANN.

However, the number of flowers on a bunch should also be considered as it determines the number of berries on a bunch, so in years that many flowers dropped, fewer berries will develop into berries on the bunches and vice versa (Vasconcelos et al., 2009). The implication of this for this study is that in the years that many flowers dropped, the bunches would have been straggly, allowing more light to interact with the other parts of the bunch and the background and not many berries during scanning. In the years when little flowers dropped, the bunches would have been compact and a lot of light would have been reflected from many more berries, but some berries would have had less surface area available for the light to interact with. In both scenarios, the fewer berries and more berries on a bunch might also play a role in the number of specific browning phenotypes that would develop, for example in the event of fewer berries for friction browning.

In addition, a table grape bunch consists of berries attached to pedicles/stems, in turn, attached to a central axis (Chervin et al., 2012). Each berry, however, acts as an individual fruit on the bunch. Different cultivars have a different number of berries on a bunch (May, 2000; Vasconcelos et al., 2009), and the size and the weight of these berries differ. ‘Regal Seedless’ table grapes can have bunches weighing up to 870, 780, and 915 g, respectively, if they contain 150 berries that each weigh 5.2 g (Van der Merwe, 2012). The application of plant growth regulators such as GA_3_ (amount, concentration, and combination as well as the time of application) would also play a major role in the size of the berries (Raban et al., 2013). The application of enlargement sprays is usually done when the berries are 4–5 mm in diameter. This enlargement of the berries can cause bunches to be compact, which leads to friction browning. The physical removal of berries and/or laterals on a bunch so that the bunch is not too compact occurs when the berries are 8–10 mm in diameter to ensure a looser bunch. Proper bunch thinning must occur so that the bunch is not too compact so that as much as possible, the surface of as many berries is exposed. This will ensure the most efficient possible collection of information of the berries and the bunch. For friction browning that is mostly concentrated at the pedicel part of the berries, the light would not always fall completely on those parts since they are obscured by the other berries.

## Materials and Methods

### Harvest Locations

‘Regal Seedless’ table grape bunches (*Vitis vinifera* L.) bunches were harvested from two different vineyard blocks, one in the Hex River Valley and one in Wellington, Western Cape, South Africa during 2016. The global positioning system (GPS) coordinates for the Wellington vineyard is 33°38′22,0″S,10°50′47,6″E and that of the Hex River Valley is 33°27′53,9″S, 19°39′43,7″E. The harvest of the ‘Regal Seedless’ from the Hex River Valley was at an average soluble solids content (SSC) level of 19.55°Brix. The average titratable acidity (TA) level was 4.17 g/L, the average pH level was 3.78, and the average SSC/TA ratio was 46.22. For the ‘Regal Seedless’ from Wellington, the values were 15.72°Brix, 4.03 g/L, 3.87, and 40.16, respectively.

### Vineyard Treatments and Harvesting of Bunches

The standard protocol for preparing table grapes for export was followed (Van der Merwe, 2012). Before the application of gibberellic acid (GA_3_) to the bunches, they were shortened and thinned by physically removing some of the berries and laterals on the bunches with scissors when the berries were 8–10 mm in diameter. This was to prevent the bunches from being too compact at the ripening and harvesting. The table grape industry uses closed-top, corrugated fiberboard cartons to pack table grapes. The dimension of these fiberboard cartons is 300×400×127 mm and has a capacity of 4.5 kg. Bunches are placed in individual plastic carry bags before being packed in boxes lined with a 2-mm perforated, low-density polyethylene (LDPE) liner bag with which the entire carton content is eventually enclosed in. The placement of a corrugated cardboard sheet at the bottom reduces abrasion damage. A moisture-absorbing membrane and a green Uvasys^®^ sulfur dioxide (SO_2_) (Tessara (Pty) LTD, Cape Town, South Africa) generator sheet covered the grapes to control decay. This dual-phase SO_2_ generating pad contains precise concentrations and particle sizes of the active ingredient sodium metabisulfite (Na_2_S_2_O_5_). It consists of a sequence of laminated plastic membranes, each bonded by a wax layer. There is a slow-release layer between the top and middle plastic membranes and a fast-release layer between the middle and bottom membranes. An autocatalytic reaction begins in a 70% relative humidity environment whereby the sheet starts releasing a time and concentration-varying stream of SO_2_. The fast-release layer sterilizes the surface of the table grapes by discharging a large enough dose of SO_2_ over a 24–48 h period to kill and eradicate any actively growing *Botrytis cinerea* fungal spores. By releasing a low, continual dose of SO_2_ gas, concentrated enough to inhibit any superficial latent or inherent *B. cinerea* spores from growing, the slow-release layer remains active for up to 120 days^∗^. When table grapes reach the overseas markets, this defect is usually responsible for a large part of the post-harvest problems experienced with table grapes (Castillo et al., 2010; Gabler et al., 2010). Table grapes are usually harvested during the cooler parts of the day between 9 and 10 am and the same was done in this study. After each box was packed with the correct number of bunches in the vineyard, the LDPE liner bag containing the grapes and SO_2_ sheet was folded, the boxes were closed, carried out of the vineyard to the end of each row, and placed in the shade until all the other grapes were harvested. The packed boxes were loaded into an air-conditioned vehicle and transported by road to the chemical analytical laboratory of the Department of Viticulture and Oenology, Stellenbosch University, South Africa. The transport of boxes 1–7 to the Agricultural Research Council at Nietvoorbij in Stellenbosch occurred after the scanning for cold storage at 0°C. Box 1 was immediately evaluated after the first scan on the day of harvest (week 0). Box 2 was cold-stored for one week (week 1), removed, and taken back to the chemical analytical laboratory of the Department of Viticulture and Oenology, Stellenbosch University, and scanned again after a few hours. The removal of box 3 was after 2 weeks of cold storage (week 2) and box 4 after 3 weeks (week 3) of cold storage, etc. This occurred up until box 7 and the same process was followed as with box 1. The evaluation of each box (2–7) for the different defects occurred immediately after the second round of scanning.

### Near-Infrared Spectroscopic Measurements

**FIGURE 1:**
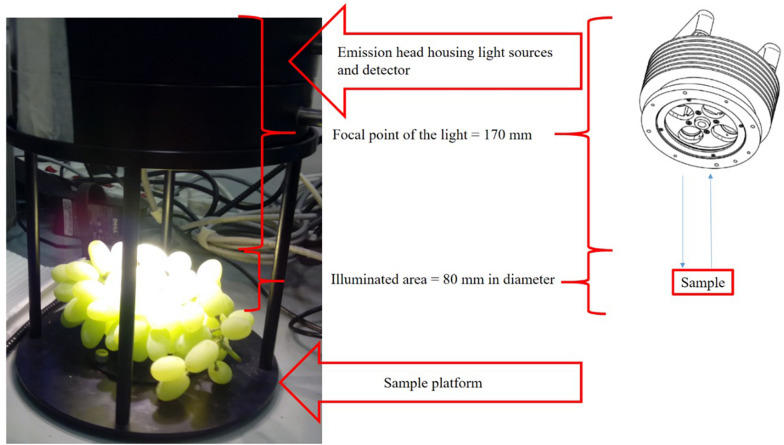
Contactless scanning of an intact ‘Thompson Seedless’ table grape bunch with the MATRIX-F NIR spectrometer (adapted from Daniels et al., 2019).

The NIR spectra of the intact table grape bunches were acquired with the diffuse reflectance MATRIX-F Fourier Transform (FT)-NIR spectrometer (Bruker Optics, Ettlingen, Germany) (Figure 1) that was operated with a contactless measurement head coupled with the spectrometer with a cable for power supply and lamp switching. Four air-cooled tungsten NIR light sources (12 V, 5 W) mounted in the measurement head illuminates the samples. The measurement area on the sample was 80 mm in diameter and the distance from the emission head to the sample plate was 170 mm. The collection of the light scattered by the sample is guided to the spectrometer with an optic fiber of 1 m. The standard viticultural practice of thinning table grape bunches ensured that the bunches were not too compact and as much as possible, the surface of as many as possible berries were exposed so that the proper information of as many of the berries on the intact bunch was collected. It is important to keep in mind that a grape bunch is not a uniform sample with many edges increasing the signal-to-noise ratio and that information collected during the scans is of all the different parts of the bunch (rachis, stems, and berries). The detecting emission head also housed a very sensitive, thermoelectric-cooled, and temperature-controlled InGaAs diode detector. Each bunch was scanned for 60 s, once in the middle of the one side and once in the middle on the other side. During each scanning procedure, 32 scans took place per side, averaged into a single spectrum. The collection of spectral data were in the range of 12,500–4,000 cm^–1^ (resolution, 2 cm^–1^; scanner velocity, 10 kHz; background, 32 scans; sample, 32 scans). The number of data points collected during each scan was 1,801. The Log (1/R) transformed absorbance spectra were processed using OPUS version 7.2 (Bruker Optics, Ettlingen, Germany) for Windows, and saved after the spectral acquisition. All the boxes were scanned immediately upon arrival in the laboratory (week 0) and then again after each week of cold storage (week 1–week 6). The storage time was, therefore, one week for each box. After each box was removed from cold storage, the LDPE liner was opened and folded over the sides of the box, the SO_2_ and moisture absorbing sheets removed and the grapes were left to acclimatize to the room temperature in the laboratory (as to be the same as the day of the initial scan) before being scanned again.

### Visual Assessment of Browning Phenotypes

A visual evaluation of each box occurred immediately after scanning e.g., the box of week 0 on the day of harvest and the other boxes after every week of cold storage (week 1–week 6). The berries removed from the bunch with scissors were individually evaluated for one type of defect only, i.e., chocolate browning or friction browning, whichever defect appears most pronounced on it. In the data collection stage, there was no recording of the status of each grape berry. Instead, the translation of the bunch was either containing the defect even if only observed on one berry or not containing the defect if all the grape berries on the bunch were clear. For ease of analysis, the bunches were assigned a value of 0 when no defect was present at all and a value of 1 when the defect was present. The incidence of chocolate browning was absent in weeks 1 and 2 of cold storage or too low in weeks 3–6 to undergo meaningful analysis. The data for the chocolate browning in weeks 3 and 4, and in weeks 5 and 6, were combined into one dataset for the data analysis. Similarly, for friction browning, the data from weeks 3 and 4 were combined, i.e., the spectra and the matching reference (presence of the browning = 1; or not = 0) were placed in one excel sheet.

### Data Analysis

#### Partial Least Squares-Discriminant Analysis

In PLS, the dummy variable Y is used as a response variable, and it is set to 1 if the sample is present and 0 if not. In this study, the defects were scored as 0 = no defect and 1 = defect present. The cut-off value was set at 0.5, above which the sample was predicted as 1, and below as 0. In this study, the optimal number of latent variables (LV) was chosen based on the minimum root mean square error of cross-validation (RMSECV). The model was cross-validated using Venetian blinds of 10 data splits with 10 samples c {[True Negatives/(True Negatives + False Positive)], sensitivity [True Positives/(True Positives + False Negatives)]}, and classification error rate (CER) for the calibration and cross-validation were also used to evaluate the performance of the model (Nicolaï et al., 2007; Ballabio and Consonni, 2013). All the calculations were performed using the PLS-Toolbox for MATLAB (version 8.6.1, Eigenvector Research Inc., United States).

#### Artificial Neural Networks

To determine the relationship between the spectral information of the studied bunches and the presence or absence of different browning phenotypes using ANN, the following procedures were followed. The relevant entries were selected (e.g., weeks in cold storage 3 and 4 from the “No defects - REGAL Week 3 and Week 4, Week 5 and Week 6” dataset). The data were normalized and labeled (0 for no defect and 1 for defect). The two combined datasets had a total of 192 samples, which were divided into four sets. Resulting in 96 training samples (∼1/2), 32 validations 1 sample (∼1/6), 32 validations 2 samples (∼1/6), and 32 testing samples (∼1/6). A maximum of four hidden layers was selected and the number of nodes (with a max of 25) in each layer and alpha was set independently. The optimal parameter combination was selected *via* a grid search (running the model for each set of parameters). The dataset dimensions (the number of wavenumbers) were reduced so that the number of dimensions was less than the number of samples, using principal component analysis (PCA).

Cohen’s kappa is a statistic (Cohen, 1960, 1968) that indicates how well a classification model does in comparison to predicting just the average (McHugh, 2012). The interpretation of the kappa score that was used is displayed in Table 1. If the kappa score was between 0 and 0.2, there was no agreement between the measured and predicted label, a score between 0.21 and 0.39 showed minimal agreement, and a score between 0.4 and 0.59 showed weak agreement. There was a moderate agreement when the kappa score was between 0.6 and 0.79. A kappa score between 0.8 and 0.9 corresponded to a strong agreement between the measured and predicted labels while a score above 0.9 showed an almost perfect agreement. In this study, a kappa score indicating a strong agreement was considered “good” and almost perfect was considered to be “great”.

**TABLE 1:** Kappa score interpretation guide (McHugh, 2012).

**Kappa**	**Interpretation**
0.0–0.20	No agreement
0.21–0.39	Minimal agreement
0.40–0.59	Weak agreement
0.60–0.79	Moderate agreement
0.80–0.90	Strong agreement
>0.90	Almost perfect agreement

## Results and Discussion

### Partial Least Squares-Discriminant Analysis

**FIGURE 2:**
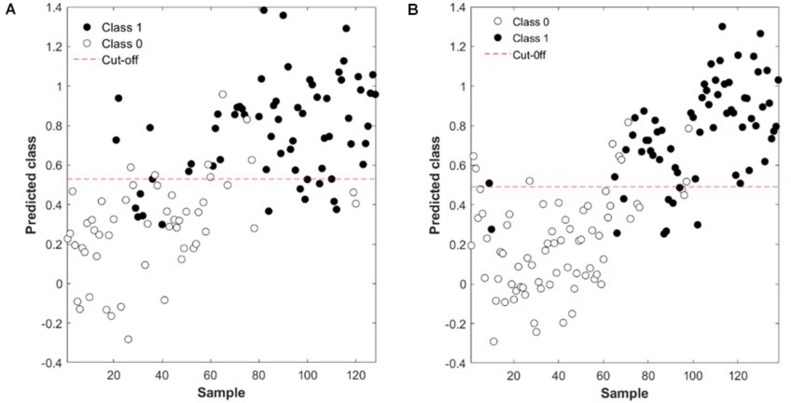
Absence (Class 0, open circle) or presence (Class 1, close circle) of chocolate browning in ‘Regal Seedless’ table grape bunches performed by a partial least squares discriminant analysis (PLS-DA) model, based on near-infrared (NIR) spectral data. **(A)** Weeks 3 and 4 and **(B)** Weeks 5 and 6.

The CER of chocolate browning for weeks 5 and 6 was lower (22%) than that of weeks 3 and 4 for both class 0 and class 1 (Table 2). This means that the prediction of chocolate browning could have an accuracy of 75% for weeks 3 and 4, and 78% for weeks 5 and 6. This might be attributed to the longer times that the samples of weeks 5 and 6 were in cold storage and the defect, therefore, developed and/or appeared more pronounced on the bunches. The incidence could also have been more (more chocolate brown berries) in weeks 5 and 6 than in weeks 3 and 4. This was also observed where the specificity is concerned since it was better for class 0 at weeks 3 and 4 (81%) and better for class 1 in weeks 5 and 6 (82%). The sensitivity, on the other hand, was higher for class 0 in weeks 5 and 6 (82%) and for class1 in weeks 3 and 4 (81%) (Figures 2A,B).

**TABLE 2:** The classification error rate, specificity, and sensitivity of the partial least squares discriminant analysis (PLS-DA) models constructed for chocolate browning (CB) and friction browning (FB) of ‘Regal Seedless’ grape bunches.

**Condition**	**Sample set**	**Class 0**			**Class 1**		
		**CER^a^**	**Spec^b^**	**Sen^c^**	**CER**	**Spec**	**Sen**
CB: W3and4^d^	Calibration	0.15	0.865	0.815	0.15	0.815	0.865
CB:W3and4	CV^e^	0.25	0.808	0.692	0.25	0.692	0.808
CB: W5and6^f^	Calibration	0.13	0.875	0.864	0.13	0.864	0.875
CB: W5and6	CV	0.22	0.722	0.818	0.22	0.818	0.722
FB: W3and4	Calibration	0.41	0.412	0.757	0.41	0.757	0.412
FB: W3and4	CV	0.46	0.353	0.714	0.26	0.714	0.353

*^*a*^Class error rate is defined as the proportion of instances misclassified over the whole set of instances.*

*^*b*^Specificity is defined as the ability of a test to correctly identify a sample without the defect.*

*^*c*^Sensitivity is defined as the ability of a test to correctly identify a sample with a defect.*

*^*d*^Weeks 3 and 4.*

*^*e*^Cross-validation.*

*^*f*^Weeks 5 and 6.*

For friction browning, the CER for class 1 (26%) was lower than that of class 0 (46%) and almost similar to that of class 0 and class 1 with chocolate browning for weeks 3 and 4 (25%) (Table 2). Both the specificity and sensitivity of class 0 and class 1 for friction browning were not as good as that for chocolate browning.

It should also be noted that a grape bunch is not a uniform sample with many edges and that berries are sticking out in all directions, increasing the signal-to-noise ratio. The information that was thus collected during the scans was of all these different parts of the bunch (berries and stems). All this might have played a role in the CER as well as the specificity and sensitivity of classes 0 and 1 obtained for friction and chocolate browning. The error rates in Haff et al.’s (2013) study when they developed an algorithm to identify spots generated in hyperspectral images of mangoes infested with fruit fly larvae were much lower than the ones in this study. They achieved an overall error rate of 2.0%, with 1.0% false positive and 3.0% false negative. This is also similar to the studies of Leemans et al. (2002) when they classified two different apple cultivars using machine vision. Their proposed method for apple external quality grading showed correct classification rates of 78 and 72%, for the Golden Delicious and Jonagold apples, respectively. When they considered the two classes (fruit accepted or rejected), the error rate reached 5% for Golden Delicious and 8% for Jonagold. Li et al. (2016) obtained a total accuracy of 96.6% when they looked at the skin defects of bi-colored peaches. Their proposed multispectral algorithm was effective in differentiating normal and defective bi-colored peaches. All these good results obtained by these different authors might be due to the larger size of mangoes, apples, and peaches that they used in their experiments and, therefore, the larger surface area that was available to the NIR light than the surface area of the single berries that was affected with the browning disorder in this study.

**FIGURE 3:**
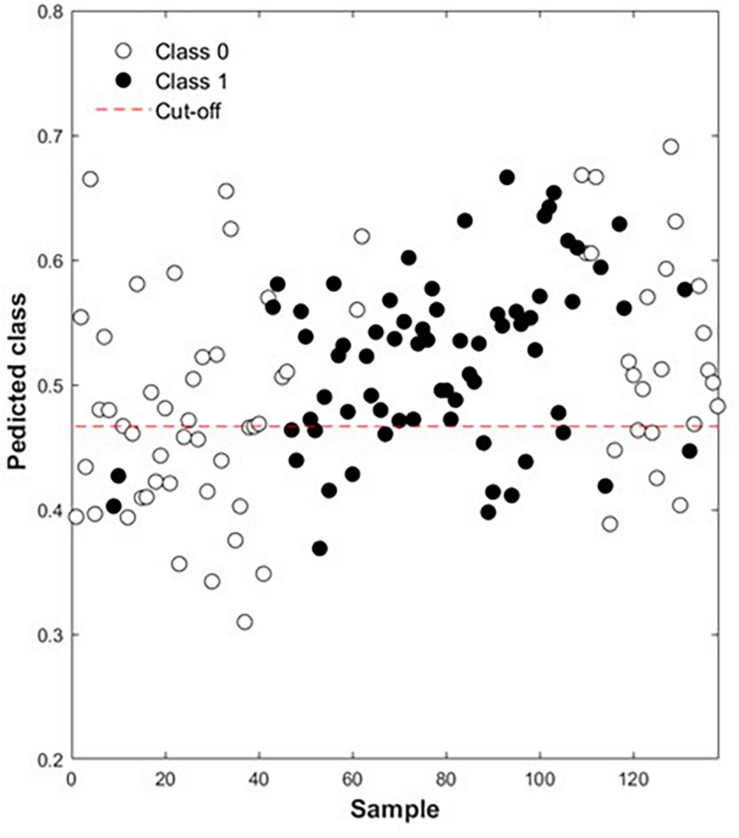
Absence (Class 0, open circle) or presence (Class 1, close circle) of friction browning in ‘Regal Seedless’ table grape bunches performed by PLS-DA model, based on NIR spectral data for Weeks 3 and 4.

Taking into account that using a value of 0 to indicate the absence of browning on the entire bunch and a value of 1 to indicate the presence there-of, even when only one berry on a bunch had browned, might not have been the most accurate way to obtain the reference for building the classification models. However, it should be kept in mind that the browning phenomenon is complex and does not occur instantaneously, but rather, gradually, as time progresses. This means that it is possible that the brown discoloration in the bunches that scored 0 (no browning) could have developed at a later stage. Similarly, where only one berry developed browning and the whole bunch was scored 1 (browning present), the affected berry could have remained the only one to develop browning, while the other berries could have remained healthy for a considerable time during cold storage. False positives and negatives would therefore be present and the accuracy of the models would thus not be completely dependable. Figure 3 illustrates the situation where the majority of bunches that should seemingly be clear are indicated as having friction browning. The spectra picked up other browning phenotypes already present on those bunches, but not yet visually discernible by the naked eye, hence the misclassification of mostly clear (class 0) bunches as class 1 bunches.

**FIGURE 4:**
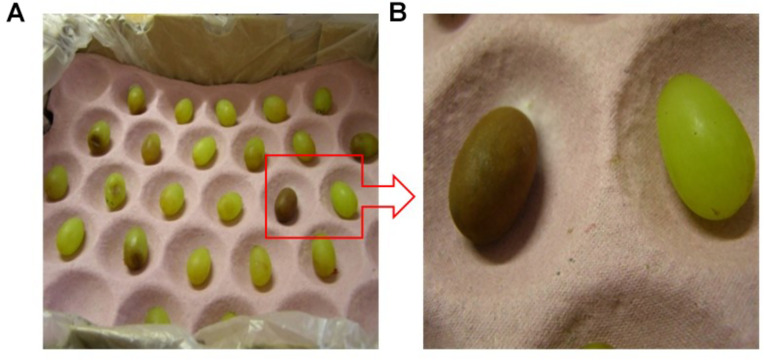
**(A)** The browning stage of berries in a peach tray after five weeks of cold storage and **(B)** a berry showing chocolate browning (caused by a fungal infection) and a berry not showing any browning.

**FIGURE 5:**
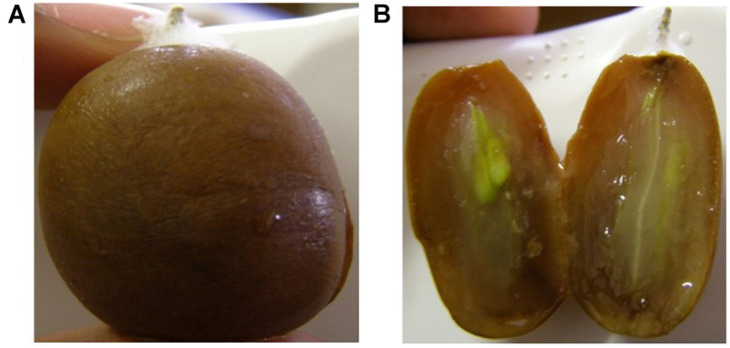
A Regal berry showing chocolate browning on the outside **(A)** and **(B)** on the inside caused by a fungal infection.

**FIGURE 6:**
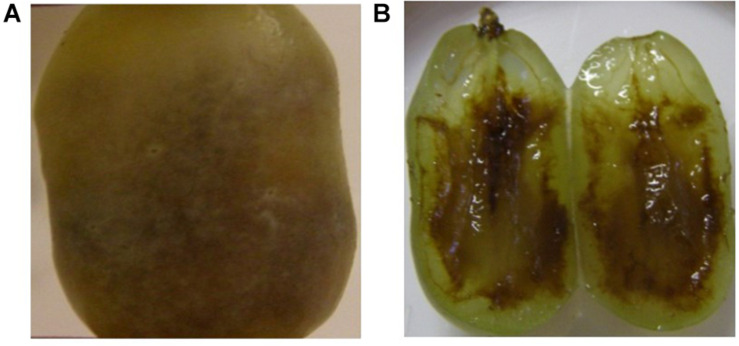
Internal browning as seen **(A)** from the outside and **(B)** on the inside of a ‘Thompson Seedless’ berry.

**FIGURE 7:**
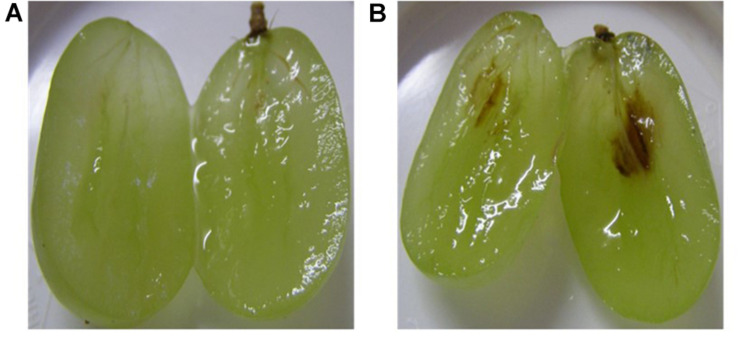
A ‘Thompson Seedless’ berry is showing **(A)** no signs of browning on the inside and **(B)** onset of internal browning around the vascular tissue.

Figures 4A,B show the complexity of browning development during cold storage with two adjacent berries in the peach tray coming from the same bunch harvested from the same vineyard in the same year. One is still healthy and clear (Figure 4B on the right) and the other has turned completely brown (Figure 4A on the left) with the possible cause of this browning being a fungal infection. Figure 5 shows the same berry in Figure 4B on the left with chocolate browning symptoms on the outside of the berry (Figure 5A) and when it is cut open (Figure 5B). Figure 6A shows internal browning symptoms as seen from the outside and Figure 6B as seen on the inside when the berry is cut open. Figure 7A shows the gradual development of internal browning from completely clear, to symptoms starting to manifest in Figure 7B.

### Artificial Neural Networks

In Table 3, the chocolate browning vs. no defects for the ‘Regal Seedless’ table grapes weeks 3 and 4 showed that there was strong agreement (test kappa = 0.88) between the measured and predicted labels for the data when PCA was performed and the number of features (wavenumbers) was reduced to 50 (three runs). From two to four runs it can be seen that accurate prediction can be done with a moderate agreement (Table 1), but not necessarily consistently (test kappa = 0.71 with two runs and 0.67 with four runs). This might be due to a lack of data causing an insufficient representation of healthy and damaged spectra variation in the training sample set. The chocolate browning vs. no defects for the ‘Regal Seedless’ table grape in weeks 5 and 6 showed a strong agreement between the measured and predicted labels for the data when PCA was performed and the number of features (wavenumbers) was reduced to 30 (two and three runs) and 15 (four runs), respectively. The test kappa scores were all above 0.80. The friction vs. no defects for the ‘Regal Seedless’ table grapes in weeks 3 and 4 showed that there was a moderate agreement between the measured and predicted labels for the data when PCA was performed and the number of features was reduced to 80 (two runs).

**TABLE 3:** The number of runs done for each cold storage condition (week 3 and week 4; week 5 and week 6) and browning defect, chocolate browning (CB), and friction browning (FB) of the ‘Regal Seedless’ table grape bunches.

**Condition**	**Runs**	**PCA**	**Alpha (α)**	**Validation kappa score**	**2nd validation score**	**Test kappa score**
CB: W3and4^a^	1	n/a	0.01	1.0	0.65	0.59
	2	80	0.1	1.0	n/a	0.71
	3	50	1e–5	1.0	0.656	0.88
	4	50	1e–6	0.93	n/a	0.69
CB: W5and6^b^	1	n/a	1e–3	1.0	0.47	0.68
	2	30	1e–5	1.0	1.0	0.83
	3	30	1e–7	1.0	1.0	0.80
	4	15	1e–6	1.0	1.0	0.83
FB: W3and4^a^	1	n/a	1e–2	0.78	n/a	0.37
	2	80	1e–2	1.0	n/a	0.73
	3	50	1e–5	1.0	0.49	0.51
	4	65	1e–9	0.92	0.77	0.47

*The number of feature reductions done per principal component analysis (PCA) and the kappa scores for the first and second validations, as well as the test validation, are also shown.*

*^*a*^Weeks 3 and 4.*

*^*b*^Weeks 5 and 6.*

In other studies where ANN was utilized as an analysis technique, Zarifneshat et al. (2012) successfully evaluated it as an alternative technique to predict the bruise volume of apples in a fast, yet accurate and objective way. Binetti et al. (2017) used it to create models to classify olive oil cultivars based on multiple types of information, standard merceological parameters, NIR data, and nuclear magnetic resonance (NMR) fingerprints. The most informative variables about the cultivars were obtained with the NMR data because the ANN models based on the NMR data displayed the highest ability to classify cultivars (in some cases, accuracy >99%), independently on the olive oil production process and year.

## Conclusion

This study shows the possibility of detecting the presence or absence of different browning phenotypes during different cold storage periods on intact ‘Regal Seedless’ table grape bunches through contactless and non-destructive scanning using NIR spectroscopy. This, coupled with PLS-DA analysis and the machine learning technique ANN, showed that chocolate browning classification is more accurate than friction browning with both techniques. Table grape bunches are very complex and heterogeneous, and the difficulty of building accurate classification models for the different browning phenotypes is also highlighted here. In the global context where consumer preference dictates the market, it is paramount that producers have an idea of what the post-harvest quality of their products is going to be. Real-time objective measurements, such as the contactless measurement of intact table grape bunches in the pack-house with NIR spectroscopy using classification models such as those constructed in this study, present a feasible alternative to exporting table grapes that might turn brown before or when they reach the intended export market. Such measures may help producers to make appropriate marketing decisions, especially for developing countries that operate in a highly competitive niche market and cannot afford any post-harvest losses.

## Data Availability Statement

The datasets presented in this study can be found in online repositories. The names of the repository/repositories and accession number(s) can be found below: http://www.kaggle.com/dataset/a10c8df9fc8f5b6c7b3ecd400878c7e0cee0d9ad0e0e5e296c5e20bf074d3ac7.

## Author Contributions

UO, HN, and AD conceptualized the research. AD conducted the experiments. AD, CP-E, and NB made the data analyses. AD, UO, CP-E, and HN wrote the manuscript. UO and HN revised and edited the manuscript.

## Conflict of Interest

The authors declare that the research was conducted in the absence of any commercial or financial relationships that could be construed as a potential conflict of interest.

## Publisher’s Note

All claims expressed in this article are solely those of the authors and do not necessarily represent those of their affiliated organizations, or those of the publisher, the editors and the reviewers. Any product that may be evaluated in this article, or claim that may be made by its manufacturer, is not guaranteed or endorsed by the publisher.
